# MicroRNA Processing and Human Cancer

**DOI:** 10.3390/jcm4081651

**Published:** 2015-08-21

**Authors:** Masahisa Ohtsuka, Hui Ling, Yuichiro Doki, Masaki Mori, George Adrian Calin

**Affiliations:** 1 Department of Experimental Therapeutics, The University of Texas, MD Anderson Cancer Center, 1881 East Road, Unit 1950, APT 1125, Houston, TX 77030, USA; E-Mails: MOhtsuka@mdanderson.org (M.O.); HLing@mdanderson.org (H.L.);; 2 Department of Gastroenterological Surgery, Osaka University Graduate School of Medicine, Yamadaoka 2-2, Suita, Osaka 565-0871, Japan; E-Mails: ydoki@gesurg.med.osaka-u.ac.jp (Y.D.); mmori@gesurg.med.osaka-u.ac.jp (M.M.)

**Keywords:** MicroRNAs, biogenesis, cancer

## Abstract

MicroRNAs (miRNAs) are short non-coding RNAs of 20 to 25 nucleotides that regulate gene expression post-transcriptionally mainly by binding to a specific sequence of the 3′ end of the untranslated region (3′UTR) of target genes. Since the first report on the clinical relevance of miRNAs in cancer, many miRNAs have been demonstrated to act as oncogenes, whereas others function as tumor suppressors. Furthermore, global miRNA dysregulation, due to alterations in miRNA processing factors, has been observed in a large variety of human cancer types. As previous studies have shown, the sequential miRNA processing can be divided into three steps: processing by RNAse in the nucleus; transportation by Exportin-5 (XPO5) from the nucleus; and processing by the RNA-induced silencing complex (RISC) in the cytoplasm. Alteration in miRNA processing genes, by genomic mutations, aberrant expression or other means, could significantly affect cancer initiation, progression and metastasis. In this review, we focus on the biogenesis of miRNAs with emphasis on the potential of miRNA processing factors in human cancers.

## 1. Introduction

MiRNAs are small non-coding RNAs of 20 to 25 nucleotides that do not code for proteins, but regulate their expression levels via post-transcriptional regulation. The canonical mechanism of miRNA action is through interaction with 3′UTR based on sequence complementarity. Recent studies suggest a much diverse mechanism of miRNA action mechanisms, including binding to the 5′UTR or the coding region with functional consequences [1]. Nonetheless, the interaction between miRNAs and their target genes changes protein output by either affecting mRNA stability or affecting protein translation. Importantly, absolute sequence complementarity between the miRNAs and their target messenger RNAs (mRNAs) is not necessary; this flexibility implies that each miRNA could bind and regulate numerous mRNAs [2,3]. It has been estimated that miRNAs regulate about 50% of all protein coding genes in mammals [4,5,6].

The first miRNA, lin-4, was discovered in *Caenorhabditis elegans* in 1993 [7]. However, the connection of miRNA to human cancer was not appreciated until 2002, when George Calin and Carlo Croce revealed that the deletion miR-15a/16-1 in chromosome 13q14 is associated with chronic lymphocytic leukemia (CLL) [8]. Subsequently, a huge number of miRNAs have been reported as “oncomiRs” or “tumor suppressive miRNAs”. For instance, the miR-17–92 cluster was identified as an oncomiR that is overexpressed in several types of B-cell lymphomas and accelerates tumor development in a c-myc-driven murine model [9,10]. On the contrary, the let-7 family is reported to have tumor suppressive effects. For example, ectopic ovderexpression of let-7 in pancreatic cancer cells with low let-7 expression blocked phosphorylation and activation of STAT3, and reduced the growth and migration of the cancer cells [11]. 

Dysregulation of miRNAs has been found in almost all human cancer types, and the aberrant miRNA expression can be caused by genomic deletion, transcription regulation, and miRNA processing. While factors such as genomic deletion and transcription regulation tend to change single or a small group of miRNAs, defects in miRNA processing machinery usually lead to broad changes at a larger scale. Early in 2006, Lu *et al.* performed miRNA profiling of 217 mammalian miRNAs and reported a general down-regulation of miRNAs in cancer, indicating a possible defect in miRNA processing [12]. Several other reports showed processing defects in the step from pre-miRNAs to mature miRNAs for several miRNAs, including miR-125b and miR-26b in human thyroid anaplastic carcinoma [13,14,15,16]. Additionally, several reports have shown that the main regulators of miRNA maturation are aberrant expressed in cancer [17,18,19]. More recently, Wegert *et al.* and Walz *et al.* demonstrated the essential role of DGCR8 (DiGeorge syndrome critical region 8) and Drosha, two key factors in miRNA processing, in carcinogenesis of Wilms tumors [20,21]. Thus, it is of particular importance to understand the involvement of miRNA biogenesis defects in human cancer for exploring novel cancer biomarkers and novel anticancer targets. 

The research on miRNA processing is rapidly evolving. In this review, we focus on factors and mechanisms that regulate miRNA biogenesis, and discuss the relevance of such alterations in human cancer. 

## 2. MicroRNA Processing Machinery

**Figure 1:**
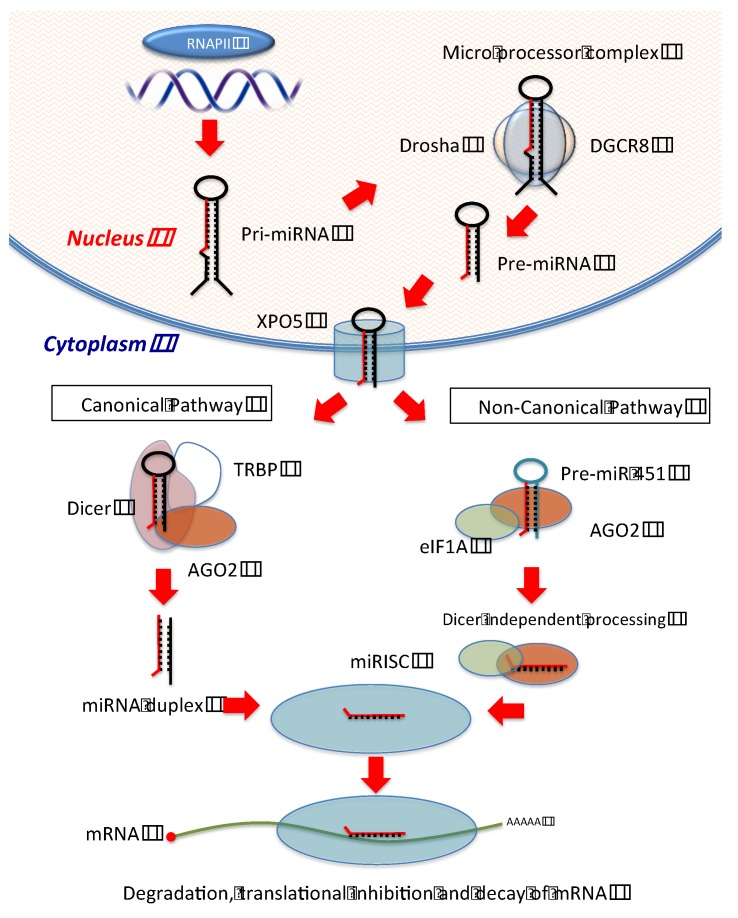
MiRNAs Biogenesis Pathway; Canonical Pathway; First, miRNAs are transcribed from the genome by RNA polymerase II (RNAP II) into primary transcripts (pri-miRNA) in the nucleus. Second, pri-miRNAs are cleaved into precursor miRNAs (pre-miRNAs) by the ribonuclease (RNAase) III family enzyme Drosha, which forms a micro-processor complex with the DNA-binding protein DGCR8. Third, miRNA precursors (pre-miRNAs) are exported to the cytoplasm by a Ran-GTP-dependent dsRNA-binding protein, Exportin-5 (XPO5). Fourth, Dicer, an RNAase III-type endonuclease, together with transactivating response RNA-binding protein (TRBP) and Kinase R-activating protein (PACT), cleaves pre-miRNAs in the cytoplasm. Finally, the guide miRNA generated by Dicer is loaded onto the RNA-induced silencing complex (RISC) consisting of Dicer, TRBP and PACT and Argonaute 2 (AGO2) and consequently binds to the 3′UTR of target genes, inducing degradation or translational inhibition of the target mRNA. Non-canonical Pathway (Dicer independent); Following transportation from nucleus to cytoplasm, pre-miR-451 is directly assembled onto AGO2-eIF1A complex. Consequently, the pre-miR-451 hairpin structure is cleaved by the Argonaute RNAase H-like motif to form the single strand mature miR-451. The generated mature miR-451 binds to the 3′UTR, and regulate expression of target genes.

The processing of miRNAs includes multiple steps that initiate in the nucleus and complete in the cytoplasm. First, miRNAs are transcribed from the genome by RNA polymerase II (RNAP II) into primary transcripts (pri-miRNA) that contain a stem-loop structure [22]. Second, pri-miRNAs are cleaved into precursor miRNAs (pre-miRNAs) with hairpin structures by the ribonuclease (RNAase) III family enzyme Drosha, which forms a micro-processor complex with the DNA-binding protein DGCR8 [23]. Previous research showed that the double-stranded stem structure and the unpaired flanking regions of pri-miRNAs are essential for binding and cleavage by DGCR8 and Drosha. DGCR8 binds to pri-miRNAs and the central part of the Drosha protein, ensuring a correct assembling. Drosha has two RNAase III domains (RIIIDs), which cleave the 3′- and 5′-strand of the stem-loop structure of miRNAs, respectively to create pre-miRNAs [24,25]. Third, pre-miRNAs are exported to the cytoplasm by a Ran-GTP-dependent dsRNA-binding protein, XPO5 [26,27]. Fourth, Dicer, an RNAase III-type endonuclease, together with transactivating response RNA-binding protein (TRBP) and Kinase R-activating protein (PACT), cleaves pre-miRNAs in the cytoplasm. The cleavage by Dicer generates a 20–25 nucleotide miRNA duplex consisting of a guide (referred to as miRNA) and passenger (referred to as miRNA*) strand [28,29]. The guide miRNA generated by Dicer is loaded onto RISC, consisting of Dicer, TRBP and PACT, Argonaute 2 (AGO2) and GW182/TNRC6 [28,30]. The RISC-miRNA complex (named miRISC) functions as a guide to detect the 3′UTR of target genes [31]. This process induces degradation or translational inhibition of the target mRNA, depending on the degree of complementarity between miRNA and its mRNA targets [32]. Generally, miRNA from the passive strand (miRNA*) is degraded and exhibits no effect on gene regulation. However, recent studies have shown that miRNA*s can also associate with the RISC complex and subsequently repress target mRNAs with biological effects similar to that of mature miRNAs [33]. Therefore, biogenesis of miRNAs is tightly controlled at multiple levels, and any defects in such processes could have important biological effects in cancer (Figure 1).

## 3. Pri- to Pre-miRNA Processing in Cancer

The genes encoding microRNAs are first transcribed by RNAP II into pri-miRNAs, which are further processed by the micro-processor complex including Drosha and DGCR8 to generate pre-miRNAs. Dysregulation of DGCR8 and Drosha have been reported to play significant roles in cancer. Deletion of DGCR8 causes DiGeorge syndrome, also known as 22q11.2 deletion syndrome, the symptoms of which include hypokalemia (due to hypoparathyroidism), immune dysfunction (due to thymic hypoplasia) and congenital heart disease [34]. In mice, deletion of DGCR8 blocks stem cell development in its early stage and decreases cell proliferation [35]. Aberrantly high expression of DGCR8 is also observed in cancer. Kim *et al.* revealed that DGCR8 mRNA expression is significantly increased in colorectal cancer compared with adjacent, histological normal tissue [36]. Conversely, down-regulation of DGCR8 enhances cellular transformation and tumor growth in lung cancer [19]. Indeed, both upregulation and downregulation of Drosha have been reported in human cancers [19,37,38,39,40]. Because the expression pattern of DGCR8 and Drosha in cancer is still controversial, further studies are necessary to elucidate the mechanisms whereby their expression patterns influence cancer pathways related to miRNA processing in the nucleus.

The recognition of the stem-loop structure of pri-miRNAs by DGCR8 is the first step in miRNA biogenesis and Alarcon *et al.* contributed immensely to our knowledge of this process [41]. They focused on RNA methyltransferase-like3 (METTL3), as it is enriched in pri-miRNA sequence in contradiction to pre-miRNA sequence. METTL3 methylates pri-miRNAs (m^6^A), which marks them for recognition and processing by DGCR8. This indicates that METTL3 might influence the expression of oncomiRs and tumor suppressive miRNAs.

RNA-binding proteins (RBPs) are closely associated with miRNA biogenesis, because they regulate each step of miRNA processing, localization and degradation. For instance, p68 (DEAD-box5 (DDX5)) and p72 (DDX72) are well known RBPs that are highly expressed in several types of human cancer [42,43]. In the nucleus, both p68 and p72 are essential for miRNA processing by Drosha, as knocking out either of these decreases the efficiency of miRNAs processing [44]. Furthermore, the Drosha complex associates with p68, which promotes conversion of pri-miRNAs to pre-miRNAs. Meanwhile p68 is related to the well-known tumor suppressor p53 and inactive p53 mutants interfere with functional assembly between the Drosha complex and p68, thereby inhibiting miRNA processing [45,46]. MiR-21, a well known oncomiR, is one of the miRNAs regulated by p68 through transforming growth factor-β (TGFβ) and bone morphogenetic protein (BMP)-specific SMAD signaling. Once p68 is down-regulated, pre-miR-21 and mature miR-21 expression is abolished, although pri-miR21 expression does not change significantly, indicating that p68 has a crucial role in miR-21 biogenesis in its TGF-β/BMP-regulated synthesis. Similarly, SMAD binds directly to the stem region of TGFβ/BMP-regulated miRNAs, thereby indirectly regulating gene expression via its regulation of miRNA maturation [47,48]. Furthermore, SMAD nuclear interacting protein 1 (SNIP1), a human forkhead-associated domain-containing protein and inhibits SMAD4, is revealed to directly combine with Drosha and regulate miRNAs processing in the nucleus by immnoprecipitation [49].

Recent studies revealed that KH-type splicing regulatory protein (KSRP) plays an important role in miRNAs biogenesis by regulating Drosha. Zhang and colleagues studied the association between this regulation system and DNA-damage response. They revealed that ATM, a regulator of DNA double-strand breaks [50,51,52], binds and phosphorylates KSRP directly as a DNA damage response, which promotes the interaction of KSRP and pri-miRNAs. Consequently, the processing of pri-miRNAs by Drosha is increased, suggesting that DNA damage pathway is associated with miRNAs biogenesis [53]. Similarly, they reported DDX1, one of the Drosha associated polypeptides, promotes pri-miRNAs maturation in ovarian cancer. After DNA damage, the ATM phosphorylation reinforces the connection of DDX1 and DDX dependent pri-miRNAs (miR-200a, miR-29c, miR-141 and miR-101), inducing cleavage of these pri-miRNAs by Drosha. These miRNAs were reported to be related to a mesenchymal feature in serous ovarian cancer [54], suggesting that DDX1 might regulate the progression of ovarian cancer [55]. As described above, DNA damage response affects multidirectionally miRNAs maturation by Drosha. 

Most recently, two study groups showed that mutations in DGCR8, Drosha, together with mutations in SIX1/2, are associated with blastemal type Wilms tumors (WT) [20,21]. Walz *et al.* reported decreased expression of mature let-7 in tumors with mutations or copy number loss of the microRNA processing genes (miRNAPGs) DGCR8 and Drosha, although the expression of primary let-7a was higher in the miRNAPG mutant group compared with the non-mutant group. This decrease in let-7a is consistent with previous reports that associate let-7a deletion with WT development through the regulation of LIN28, an RBP [56,57]. Interestingly, the miR-200 family (miR-200a, -200b, -141 and -429), which is associated with Mesenchymal-Epithelial Transition (MET) and stem cell maintenance is also decreased following mutations in miRNAPGs. MET induces renal structural formation during early development and blocking MET by down-regulation of the miR-200 family is thought to increase undifferentiated cells, consequently promoting WT development [21]. Wegert *et al.* also mentioned that all miRNAs evaluated by the microarray are significantly decreased in the mutant Drosha group, although there is no clear study showing that Drosha mutation induces global miRNAs dysregulation in WT [20]. These studies substantiate the importance of DGCR and Drosha in miRNAs processing in cancer (Table 1).

## 4. MiRNA Transportation and Cancer

The transporter protein XPO5 transfers pre-miRNAs from the nucleus to the cytoplasm via the small GTPase Ran. In the nucleus, XPO5 and pre-miRNAs bind to RanGTP and this complex is then transported to the cytoplasm where pre-miRNAs are released by the RanGAP-induced hydrolysis of RanGTP to RanGDP.

In cancer, this transportation process can be disrupted by several factors. Melo *et al.* reported that XPO5 mutation constrains pre-miRNAs to the nucleus, thereby preventing miRNA maturation. In XPO5-mutant cancer cells, XPO5 transfection increases expression of the miR-200 family, let-7a and miR-26a (recognized tumor-suppressors), indicating that XPO5 has tumor-suppressive features [58]. Additionally, mutant XPO5 lacks the *C*-terminal region that facilitates binding of pre-miRNAs to XPO5 and RanGTP, which leads to accumulation of pre-miRNAs in the nucleus [59]. Interestingly, Li *et al.* showed that miR-138 regulates XPO5 stability by regulating required for meiotic nuclear division the expression of 5 homolog A (RMND5A). MiR-138 is associated with tumor progression, metastasis and cell differentiation in Hela cells. Additionally, in neck squamous cell carcinoma, miR-138 decreases the downstream E-cadherin gene (CDH1) and influences EMT by altering expression of EZH2, VIM and ZEB2 [60]. As expected, miR-138 is also processed by XPO5, but miR-138 represses the stability of XPO5 and decreases miRNA processing, indicating the existence of a feedback loop in the miR-138/RMND5A/XPO5 pathway [61]. In contrast, DNA damage accelerates miRNA processing through the ATM-AKT pathway. During the DNA damage response, ATM phosphorylates effector proteins and induces DNA-damage signaling. Once AKT is phosphorylated and activated by ATM, Nup153 (a nucleopore) is phosphorylated and binds to XPO5, which induces nuclear export of pre-miRNAs [62] (Table 1).

## 5. Pre-miR to Mature MiRNA Processing and Cancer

Dicer belongs to the Ribonuclease III family of nucleases and contains Piwi/Argonaute/Zwille (PAZ) domains that bind the 2-nt 3′ overhang of dsRNA, inducing shortening of the dsRNA strand. Dicer has been well studied and is thought to be essential for miRNA as well as siRNA biogenesis [63,64]. 

Aberrant expression of Dicer has been reported in several types of cancer. For example, Merritt *et al.* reported that high levels of Dicer expression are associated with good prognosis in ovarian cancer, as well as lung and breast cancer [39]. In contrast, in colorectal and prostate cancer, increased expression of Dicer is associated with poor prognosis [65,66,67]. Down-regulation of Dicer is associated with poor prognosis in CLL, where unfavorable cytogenetic aberrations are more frequently found in patients with lower levels of Dicer [68]. Because of the variation in Dicer expression by cancer type, using Dicer as a biomarker in cancer remains controversial.

**Table 1:** Dysregulation of MiRNAs Biogenesis in Cancers.

Location	MiRNAs Processing Related Factors	Clinical Relevance	Referrence
Nucleus	DGCR8	Deletion of DGCR8 induces DiGeorge syndrome (22q.2 deletion syndrome).	[34]
		Deletion of DGCR8 reduces stem cell development and cell proliferation in mice.	[35]
		In colorectal cancer, DGCR8 expression is increased in tumors compared with normal tissue.	[36]
		Down-regulation of DGCR8 enhances cellular transformation and tumor gurowth in lung cancer.	[19]
	Drosha	Up-regulation of Drosha regulates cell proliferation; associated with poor prognosis of esophageal cancer and non-small cell lung cancer.	[37,38]
		Low expression of Drosha is associated with poor prognosis of ovarian cancer and neurobastoma.	[39,40]
	Mutations of DGCR8 and Drosha	Together with the mutations in SIX1/2, mutations of DGCR8 and Drosha are associated with Wilms tumor.	[20,21]
	METTL3	METTL3 regulates the recognition of stem-loop structure of pri-miRNAs by DGCR8.	[41]
	P68 and P72; RBPs	p68 and p72 are highly expressed in cancer and associated with miRNAs processing by Drosha.	[42,43,44,45,46]
	SMAD and SNIP1	By regulating Drosha, SMAD and SNIP1 block the maturation of oncomiRs.	[47,48,49]
	KSRP, DDX1; DNA damage	ATM phosphorylation regulates the binding of KSRP and DDX1 to Drosha.	[53,54,55]
Trans Nuclear Membrane	XP05	XP05 increases the expression levels of tumor-suppressor miRNAs, indicating that XP05 has tumor-suppressive features.	[58,59]
	RMND5A	RMNDA5A regulates XP05 stability together with miR-138.	[60,61]
	AKM-AKT signal; DNA damage	The activation of ATM-AKT signal after DNA damage, Nup153 binds to XP05, which induces nuclear export of pre-miRNAs.	[62]
Cytoplasm	Dicer	High levels of Dicer expression are associated with god prognosis in ovarian cancer, breast cancer and CLL.	[39]
		Up-regulation of Dicer is associated with poor progonosis in colorectal and prostate cancer.	[65,66,67]
	Mutation of Dicer	Dicer mutation incuce a Dicer-related disorders including PPB.	[69,70]
	TRBP and TARBP2	TRBP and TARBP2 destabilize Dicer, impairing miRNAs processing in human cancer.	[74,75]
	AG02	AG02 regulates Dicer independent miRNA-451 through the non-canonical pathway.	[88,89]
	EGFR	In hypoxic condition, EGFR binds to AG02 and blocks miRNAs maturation.	[90,91]

Dicer mutations influence cancer initiation and/or development. The first report of a Dicer mutation in cancer was in pleuropulmonary blastoma (PPB), a rare childhood malignancy of the lung or pleural cavity [69]. This study found loss-of-function mutations in Dicer1 in eleven PPB-affected families by DNA sequencing. They showed that this mutation induced aberrant expression of miRNAs, which promoted mesenchymal cell proliferation. Since this first report, Dicer1 mutation has been reported in several tumors, such as Sertoli-Leydig cell tumors, embryonal rhabdomyosarcoma and multinodular goiter; these disorders are now widely recognized as Dicer1-related disorders [70] (Table 1). 

## 6. RISC-Related Defects in Cancer

The RISC proteins include, but limited to Dicer, TRBP, PACT and AGO2. The double-stranded RNA (dsRNA)-binding proteins TRBP and PACT, associate with Dicer and regulate its function of miRNA biogenesis [71]. Lee *et al.* revealed that PACT is essential for accumulation of mature miRNAs, because depletion of PACT promotes miRNA maturation [72]. Although TRBP and PACT both possess dsRNA-binding domains (dsRBDs), TRBP and PACT have different roles in Dicer-related miRNA processing. DsRBD 1 and 2 (the two *N*-terminal dsRBDs of each protein) are essential for the interaction between Dicer and TRBP or PACT; differences in these domains (between TRBP and PACT) alters Dicer-dependent dsRNA substrate recognition and processing, affecting both substrate and cleavage specificity during miRNA and siRNA production [73].

Decreased expression of TRBP (induced by mutation of TAR RNA-binding protein 2 (TARBP2)) is related to a destabilization of Dicer, which impairs miRNA processing in human cancer cell lines and sporadic and heredity carcinomas with microsatellite instability [74]. Furthermore, TARBP2-dependent miRNAs, miRNA-143 and miRNA-145, restrict development and tumor growth in cancer stem cells of Ewing sarcoma family tumor [75]. These data show that TRBP acts as a tumor suppressor through its role in miRNA processing. 

Finally, AGO2 is an important component of miRISC. Argonaute protein was initially discovered in plants and is now recognized as a highly conserved protein between all species [76,77,78]. Agonaute proteins have four domains (*N*-terminal domain, PAZ domain, MID domain and PIWI domain) with two linker structures (L1 and L2) [79]. The PAZ domain binds to the 3′ end of small RNAs including miRNAs, whereas the MID domain binds to the 5′ end [80,81]. The PIWI domain plays a role in cleaving small RNAs, as this domain has an RNase-H like structure [82,83]. Of the four Argonaute proteins (AGO1–4), only AGO2 has endonucleolytic and consequent gene silencing activity against mRNAs [84,85]. Furthermore, AGO2 increases the level of mature miRNAs independently from its RNAse activity, suggesting that AGO2 also affects miRNAs maturation [86,87]. However, it has recently been reported that AGO2 affects miR-451 maturation mainly through the non-canonical pathway of miRNA biogenesis [88]. Instead of being processed by Dicer, pre-miR-451 is loaded onto AGO2 [89]; eukaryotic translation initiation factor (eIF1A) binds to the MID domain of AGO2 to form an eIF1A-AGO2 complex, promoting miR-451 maturation through the non-canonical pathway [88]. Since Dicer-independent miR-451 is an important cancer biomarker, AGO2-dependent miRNA biogenesis requires further investigation (Figure 1).

Furthermore, epidermal growth factor receptor (EGFR), a well-known oncogene, regulates miRNAs biogenesis in hypoxic condition associated with AGO2. Shen *et al.* revealed that hypoxia enhances the connection between EGFR and AGO2, which induces specific AGO-2 phosphorylaiton (AGO2-Y393 phosphorylation). As a consequence, the binding of AGO2 to Dicer is diminished, blocking miRNAs maturation in cytoplasm [90]. Conversely, Hypoxia is reported to promote miRNAs by hydroxylation of AGO2, which enhances its endonuclease activity [91]. Hence, the regulation mechanism of AGO2 is still controversial and needs further studies (Table 1).

## 7. Conclusions

Since the first discovery of miRNAs, an enormous amount of studies have focused on uncovering the clinical importance of these small RNAs. There are currently several clinical trials targeting miRNAs or using miRNA mimics [92]. Miravirsen, a locked nucleic acid-modified DNA phosphorothioate oligonucleotide complementary to miR-122, binds to two adjacent target sites in 5′UTR region of hepatitis C virus (HCV) RNA that is essential for its RNA replication [93]. Consequently, this drug decreases the expression of HCV RNA in a dose-dependent manner in the patients with HCV-induced cirrhosis [94]. In the cancer field, tumor suppressors miR-34 and let-7 have been explored as possible therapeutic agents. The clinical trial of miR-34 mimics (MRX34) against hepatocellular carcinoma and metastatic liver cancer is now in phase I (ClinicalTrials.gov Identifier: NCT01829971). Thus, miRNA-targeted therapeutics is likely to be a promising treatment option against several diseases, including cancer. Although miRNA-targeted therapy has certain obstacles challenging effective and precise conveyance to the tumor sites, with the advancement on the nucleotide delivery system, we are optimal that miRNA therapies will likely be used in treating cancer patients in the near future. Furthermore, circulating miRNAs are remarkably stable even in human body fluids, and thus have been extensively explored as cancer biomarkers. Some studies suggest that the stability of circulating miRNAs are due to membrane-bound vesicles, which envelope miRNAs and prevent degradation from RNAase [95,96,97]. Interestingly, recent study reported that exsomes (one of membrane-bound vesicles) derived from cancer tissue contains not only miRNAs, but also RISC complex which induce maturation of oncogenin miRNAs [98]. These findings indicate that circulating exosomal microRNA biogenesis factors might affect carcinogenesis and tumor progression, and thus represent unique candidates of novel cancer biomarkers. 

Conversely, mechanisms of miRNA biogenesis disregulation are relatively less well studied compared with miRNAs themselves. However, Dicer-substrate siRNAs (DsiRNAs), Dicer related gene knocking down system, are of current interest as new therapeutic tools in cancer [99,100,101,102]. Conventionally, small interfering RNAs (siRNAs) are designed as 21mer RNA duplexes and the active strand silences gene expression post-transcriptionally by binding of short strands of homologous RNA to target mRNA. On the other hand, 27mer dsiRNAs are cleaved by Dicer into 21mer siRNAs and perform better silencing than canonical siRNAs. Since siRNAs cleaved from dsiRNAs are directly connected to RISC complex, this system is thought to enhance gene knockdown efficacy [99]. Currently, two clinical trials are ongoing using DCR-MYC, which is the first MYC-targeting siRNA to enter clinical trials. A phase 1 clinical trial of DCR-MYC is being conducted in patients with solid tumors, multiple myeloma or lymphoma, and a phase 1b/2 trial in patients with hepatocellular carcinoma (ClinicalTrials.gov Identifier: NCT02110563 and NCT02314052). Thus, the new treatments related to microRNAs biogenesis factors have just started. The success of this technique indicates that the efficacy of miRNAs mimics (Double-stranded RNA oligonucleotides) could improve with similar strategy.

As numerous miRNAs are regulated by miRNA-processing-related factors, this multiplicity indicates the difficulty of applying them in a clinical setting and meanwhile includes additional potential opportunities to develop these factors as possible biomarkers and therapeutic targets. Hence, further research is necessary to unveil the comprehensive miRNA biogenesis network, which will undoubtedly lead to novel discoveries relevant to the diagnosis and treatment of cancer.
